# Interventions for Functional and Cosmetic Outcomes Post Burn for Eyelid Ectropion—A Scoping Review

**DOI:** 10.3390/ebj6030046

**Published:** 2025-08-19

**Authors:** Andrea Mc Kittrick, Lola Hammond, Jason Brown

**Affiliations:** 1 Department of Occupational Therapy, Royal Brisbane and Women’s Hospital, Herston, QLD 4029, Australia; 2 School of Health and Rehabilitation Sciences, The University of Queensland, St Lucia, QLD 4072, Australia; 3 Professor Stuart Pegg Adult Burns Centre, Royal Brisbane and Women’s Hospital, Herston, QLD 4029, Australia

**Keywords:** eyelid, ectropion, burns, contracture, citriax

## Abstract

**Rationale**: Burn injuries to the face can have devastating consequences functionally and cosmetically for individuals and can result in increased depressive symptoms and low self-esteem. Burn injuries have the potential to cause contracture of the skin, especially on the face due to multiple concave surfaces, possibly causing facial deformity. These functional and cosmetic implications can interrupt activities of daily living. Although there is consensus in the literature that early interventions contribute to improved outcomes for eyelid ectropion, there is currently limited consensus regarding the techniques used in the management of eyelid ectropion post burn injuries. **Objectives**: The aim of this scoping review was to explore the evidence in the literature to identify surgical and non-surgical techniques to manage and prevent eyelid ectropion post burn. **Method**: Five databases (PubMed, CINAHL, Embase, Cochrane, and Scopus) were searched for articles published between January 2014 and August 2024. Two reviewers completed the search. Each article was screened independently by each reviewer against the inclusion and exclusion criteria. Where disagreement arose, a third reviewer was consulted for resolution. **Results**: *n* = 56 articles were sources in the initial search. Post screening, *n* = 20 met the criteria for full review; *n* = 14 were included in the final review. All studies reported on surgical techniques used to manage eyelid ectropion post burn, and only one study reported on non-surgical techniques. All studies were observational in design. **Conclusions**: There is a paucity of research addressing the surgical and non-surgical techniques for the management and prevention of eyelid ectropion following burns in the adult population. The existing literature primarily consists of case studies and case series, which limits the robustness of the evidence base for the effective management of this condition post burn.

## 1. Introduction

Burn injuries to the face can have devastating consequences for individuals, with evidence of increased depressive symptoms and reduced self-esteem [1]. The face is a visible part of the human body and is associated with beauty in many cultures [2]. Facial features are the most recognizable aspect of a person, play a role in identity, and create feelings of familiarity [3]. Burn injuries to the face have the potential to disrupt these functions and self-identity depending on the depth of burn [1]. While not all burn depths will result in burn scars or poor outcomes, the unknown consequences in the initial stages post burn can result in psychological distress [4]. Burn scarring of the eye, specifically involving the ocular surface and adjacent structures, is a rare but severe consequence of thermal, chemical, or electrical injuries [5]. Scarring on the ocular surface can severely compromise ocular motility, tear film distribution, and eyelid function [6]. Eyelid ectropion is a frequent and challenging complication arising from burn injuries to the periorbital region [5]. It is characterized by the outward eversion of the eyelid margin, which compromises the protective function of the eyelids and exposes the ocular surface to desiccation, irritation, and potential ulceration [7]. These injuries can directly damage the globe and periocular tissues, resulting in complex healing processes that frequently lead to cicatricial changes [8]. When the ocular surface itself is involved—particularly the conjunctiva and cornea—the risks include symblepharon formation (adhesion between the eyelid and globe), corneal opacification, neovascularization, and ultimately vision loss resulting in disability [7,9]. The eye’s response to thermal injury is determined by the depth, duration, and mechanism of exposure [5]. In instances where eyelid burns accompany ocular surface injuries, the resulting contractures can exacerbate exposure keratopathy, compounding the damage [6,9]. Additionally, fibrosis of the conjunctiva and Tenon’s capsule may restrict globe movement and contribute to diplopia or ectropion [6,8].

Contractures have the potential to develop over any moveable surface and given the face is dynamic, the risk of contracture development is high [10,11]. Gravity and atmospheric downward forces impact contracture development of the skin, which can result in deformities of facial features [5]. Deformities of facial features can have both cosmetic and functional consequences and may include reduced mouth opening in both the horizontal and vertical planes, ala flaring, and development of ectropion around the eyes [5,10]. Donelan and Bojovic [5] describe these deformities in terms of two categories: Type I, diffuse or focal scarring of the face with associated contractures and Type II, pan facial burn deformities [5]. Type II is classified as the more severe of the two categories, with some or all “stigmata”, including hypertrophic scarring, contractures, pigmentation changes, and psychological and functional impacts [5].

Contracture development around the eye is particularly difficult to manage and requires in-depth understanding of aesthetic units and scar contracture patterns [5]. Contractures of the upper and lower eyelids can result in ectropion exposing the eye [6]. This has both cosmetic and functional ramifications. From a cosmetic perspective—the inner surface of the lower eyelid becomes exposed; this is red in colour and can change the position of the eyelid crease, resulting in disfigurement [6]. From a functional perspective, the blink reflex may become inefficient, resulting in irritation, dry eyes, pain, and discomfort [5]. Ultimately, if the eyelids do not function as normal, there is a risk of developing ulcers; infections; or in severe cases, interruption/loss of eyesight [5]. When treating burns to the eyelids, consideration is required to prevent these cosmetic and functional long-term deformities [11].

### 1.1. Rationale

To date, there is no consensus pertaining to the management of ectropion of the eyes post burn injury [8]. The timing of interventions is also unclear; however, there appears to be consensus in the literature that early intervention leads to improved outcomes [8]. In the instances of children who sustain burn injuries to their eyes, there is acknowledgement in the literature that they are more likely to require intensive reconstruction over time [12]. Prevention and management of upper and lower eyelid ectropion post burn can be achieved using both surgical and non-surgical techniques. Surgical management of eyelid contractures can include procedures such as tarsorrhaphy, canthopexy, or use of skin grafts [7]. Non-surgical management of eyelid contractures post burn can include scar management techniques such as massage, splinting, and taping [11]. Therefore, it is crucial to examine the current literature to identify the most up-to-date practices employed in the management of facial burn injuries, with the goal of ensuring long-term, high-quality outcomes for affected individuals.

### 1.2. Objectives

The aim of this scoping review is to identify surgical and non-surgical techniques currently utilised in the management and prevention of eyelid ectropion following burn injuries.

## 2. Methods

### 2.1. Protocol

A scoping review methodology was used to determine consensus in the literature pertaining to approaches used to treat and prevent eyelid ectropion post burn in clinical practice across the spectrum of care. This methodology was utilised as the field of burn care is rapidly changing, and as such, a broad search was necessary to provide a wide view of the current evidence. The protocol for this review was prepared using the Preferred Reporting Items for Systematic Reviews and Meta-Analysis, Scoping Review extension (PRISMA-ScR) guidelines [13]. The PRISMA-ScR checklist can be found in Appendix A.1.

### 2.2. Eligibility Criteria

The PCC (participants, concept, and context) framework of the Joanna Briggs Institute (JBI) Scoping Review Manual for Evidence Synthesis [14] was employed to define the eligibility of the research question and to determine the study inclusion criteria before the onset of the selection process for separate reviewers. Included studies involved the following: peer-reviewed studies published in English between 1 Jan 2014 and 12 August 2024; investigating treatment and/or prevention of ectropion through surgical or non-surgical treatment modalities; and inclusion of outcome measurements relating to functional deficits or cosmetic deformities related to eyelid ectropion for upper/lower eyelids post burn injury. As this review was undertaken at a burn centre which manages adults burn injuries only, this scoping review was geared toward this population specifically. Table 1 below summarises the inclusion and exclusion criteria.

### 2.3. Information Sources

To identify relevant studies, the following databases were searched in August 2024: PubMed, CINAHL Complete, Cochrane Library (Wiley), Embase, and Scopus. The search strategy was guided by an experienced research librarian and was further refined through discussion within the research team. The following keywords were used: [“eyelid”] AND [“ectropion”] AND [“burn”]. The final search strategy for each database can be found in Appendix A.2.

### 2.4. Search

All sources identified by the search within each database were collated and exported to EndNote 21™ v. 21.5 (Bld 20846). Sources were grouped by database and retrieved in full, and citation details were then imported into the reference management software Covidence™ for data extraction and review. Duplicates were automatically removed upon import to Covidence™. The electronic database search was supplemented by scanning reference lists of relevant studies.

### 2.5. Selection of Sources

To achieve rigor in the selection of resources, two reviewers (L.H. and A.J.) independently screened the title and abstract of articles to determine potentially relevant publications. The reviewers progressed to full-text review to evaluate eligibility of the remaining articles for inclusion in this scoping review. Any disagreement on study selection was discussed and resolved between the two reviewers, and further disagreement after discussion was resolved by a third independent reviewer (A.M.K.) as required. Reviewers noted the primary reason for each study exclusion using the eligibility criteria throughout the selection process.

### 2.6. Data Charting Process

The data extraction form was agreed upon by the research team prior to data collection. Two reviewers independently extracted the data; inconsistencies were discussed between the two reviewers (L.H. and A.J.), and any discrepancies were reviewed by a third reviewer (A.M.K.) and finalised. Upon data entry completion, a table summarising all included studies was created.

### 2.7. Data Items

The following data points were extracted: article characteristics (country of origin, authors, title, year of publication, journal, and study type), participant sample size, eyelid sample size, intervention type, primary mechanism of injury (flame, thermal, contact, scald, and chemical), average time from burn for participants, mean follow up time (months), specific intervention applied, and outcome measure used/results reported (including functional outcome measures such as presence of ectropion, presence of lagophthalmos, eyelid contraction, eye exposure complications/corneal exposure, complications (minor for example, irritations, and major loss of skin graft or vision), scar appearance, eyelid competence, eyelid retraction, graft take, and recurrence of ectropion, as well as cosmetic outcome measures such as cosmetic questionnaires, patient satisfaction, photographs, symmetry, and scar appearance).

### 2.8. Levels of Evidence

The Joanna Briggs Institute (JBI) Level of Evidence [15] was used to determine the strength of each study included in the review. JBI categorizes evidence across five levels (Level 1 to Level 5) for different types of questions. Each level corresponds to the rigor of the research design—with Level 1 being the strongest, and Level 5 being the weakest.

### 2.9. Synthesis of Results

Due to the variation in study design and outcome measurement, it was not possible to complete a structured analysis; however, the results are presented as a narrative synthesis for broader understanding of the treatment/prevention of eyelid ectropion in burn injuries.

## 3. Results

### 3.1. Selection of Sources of Evidence

**Figure 1:**
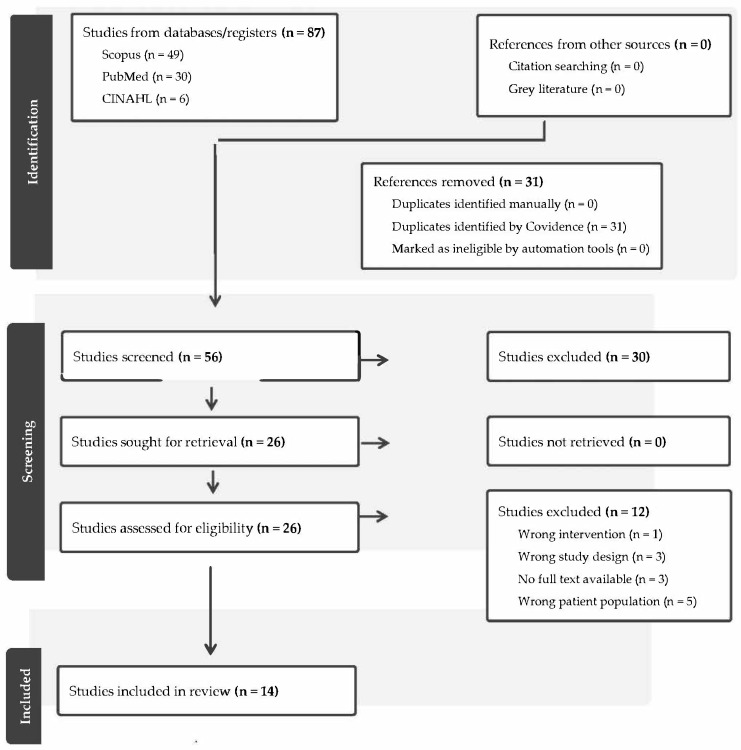
PRISMA flow chart.

The initial search yielded *n* = 87 potential studies. Post removal of duplicates, *n* = 56 studies were left for consideration. Title and abstract review identified *n* = 26 studies for potential inclusion. Following full-text review, *n* = 14 were included in the final scoping review; see Figure 1.

### 3.2. Characteristics of Studies

The characteristics of included studies and the techniques for treatment/management of eyelid ectropion are summarized in Table 2. Descriptions of the populations, interventions, outcome measures, and results of each study are outlined in Table 3. All *n* = 14 studies included in this scoping review addressed management of existing eyelid ectropion through surgical approaches using various techniques. Two studies, Clayton, Haertsch [16] and Keilani, De Faria [17], discussed early surgical techniques used to prevent the occurrence of eyelid ectropion in addition to reconstruction at a later time point. Only one study by Clayton, Haertsch [16] described non-surgical techniques for the management of eyelid ectropion post burn injury.

In this scoping review, the timing of interventions were as follows: *n* = 4 studies focused on acute intervention for management of eyelid burns at the time of initial injury [7,16,17,23], *n* = 6 studies reported reconstructive interventions for established ectropion after acute burn injury [18,19,20,21,25,28], *n* = 3 studies focused on reconstructive treatment without specification of initial interventions [22,24,26], and *n* = 1 study did not specify timing of treatment [27].

The studies included a mix of single-patient case reports and larger case series, with *n* = 1–26 participants. The most common study design was case study (*n* = 6, 42.85%), followed by case series (*n* = 3, 21.42%). The studies were conducted by institutions across different countries, including The United States of America, France, the United Kingdom, Greece, Turkey, Switzerland, Brazil, China, Egypt, Australia, Bosnia and Herzegovina, and Japan.

### 3.3. Critical Appraisal Within Sources of Evidence

Using the Joanna Briggs Institute (JBI) Levels of Evidence for Effectiveness [15], all studies included in this scoping review were determined to be Level 4 evidence; see Table 2.

### 3.4. Synthesis of Results

While most studies reported surgical techniques for management of ectropion of the eye, only one study reported non-surgical techniques. No studies specifically focused on the prevention of eyelid ectropion post burn injury in the acute or subacute phase. The most common surgical procedures reported were skin grafting (split-thickness and full-thickness grafts) [7,17,18,19,20,21,23,24,25,26,28], followed by localised flaps [7,18,19,20,24,27,28]. A wide variation of outcomes were reported, including resolution of ectropion [22,23,25], recurrence of ectropion [7,19,24,27,28], and presence of lagophthalmos [7,16,17,18,19,22,25,27,28]. Functional outcomes included measurement of eyelid function and competence [7,16,17,18,19,21,23,24,28], deficit in eyelid closure (measured in mm) [16,18,19,23,25,28], and at rest eyelid separation (measured in mm) [19,25,26]. The objective measures reported were photographs of eye closure at rest and active closure [10,23], and photographs of post-burn scarring [18,23]. Subjective outcome measures pertaining to function and appearance included subjective interview for symptoms [26] and use of satisfaction questionnaires and rating scales [17,22,26,27]. Complications were also recorded as adverse outcomes, including presence of exposure keratopathy/cornea [7,17,19,21,22,23,24,25] and percentage of graft failure rate [19,21,26]. Cosmetic outcome were least reported by symmetry of eyelids [28], cosmetic complications [25], and scar/skin appearance [22,24]. Table 3 outlines the findings for each study.

## 4. Discussion

This scoping review examined the current body of literature regarding the management of eyelid ectropion post burn injury, with an emphasis on both surgical and non-surgical interventions. Fourteen studies met the inclusion criteria, all of which described surgical management techniques, while only one study addressed non-surgical techniques. The pathophysiology of post-burn ectropion primarily involves cicatricial contracture resulting from scar formation and tissue fibrosis, leading to shortening and tightening of the anterior lamella of the eyelid [8]. This often results in lagophthalmos and exposure keratopathy, increasing the risk of corneal ulceration, infection, and subsequent visual impairment [7,8]. This is consistent with the findings of this scoping review with over half of the included studies reporting these complications, although of note, no studies in this review reported loss of vision which contrasts known consequences [8]. The severity of ectropion correlates with the depth and extent of the burn injury, with full-thickness burns presenting a higher risk due to significant tissue loss and scarring [5]. Clinical management necessitates a comprehensive approach that addresses both the functional and cosmetic sequelae, often requiring early intervention to prevent irreversible ocular damage [6].While an algorithm for management of ectropion exists [29], the complexity of burn scar formation and burn scar contracture [8] presents challenges to this algorithm.

Timely surgical intervention is essential in the management of eyelid burns to prevent both functional impairment and long-term disfigurement [5,6]. The eyelids play a critical role in protecting the ocular surface, maintaining corneal hydration, and facilitating visual function; thus, early and appropriate reconstruction is paramount [7]. In cases of deep dermal or full-thickness burns, early excision of nonviable tissue followed by grafting or flap reconstruction can reduce the risk of complications such as cicatricial ectropion, lagophthalmos, and exposure keratopathy [5,6,7]. This is consistent with the findings of this scoping review, with all studies reporting surgical techniques for the management of post-burn ectropion of the eyelid. Delays in surgical management increase the likelihood of scar contracture, leading to impaired eyelid closure and potential vision-threatening sequelae [5]. Therefore, prompt surgical assessment and interventions are vital components of comprehensive burn care to preserve both ocular function and periorbital aesthetics [5]. This evidence is consistent with the findings of this scoping review, which predominantly advocates for early surgical techniques for the management of eyelid ectropion post burn injury.

Surgical management of post-burn eyelid ectropion is a complex and often staged process aimed at restoring eyelid position, protecting the ocular surface, and improving both functional and aesthetic outcomes [6]. The choice of intervention depends on the severity of the ectropion, the extent of scar contracture, and the involvement of anterior and/or posterior lamellae [7]. Initial steps typically involve the release of scar contracture through scar excision or Z-plasty techniques to restore eyelid mobility [6]. Reconstruction of the anterior lamella is commonly achieved using full-thickness skin grafts, often harvested from donor sites with similar texture and colour, such as the upper eyelid, postauricular area, or supraclavicular region [5,6]. Eighty five percent of studies in this scoping review reported surgical techniques which aligned with this evidence base. In cases with more extensive tissue loss or poor vascularity, local flaps such as the Mustardé cheek rotation flap or Tripier flap—may be required to provide adequate tissue coverage and support [5]. For defects involving the posterior lamella, tarso conjunctival grafts, hard palate mucosa grafts, or composite grafts may be utilized to re-establish structural integrity [5]. The frequency of using local flaps as a surgical technique was reported for only forty-three percent of studies included in this review. This may be attributed to multiple factors, including availability of donor sites and burn depths of participants in the included studies. However, due to the design of the included studies, this could not be conclusively determined.

Lateral canthoplasty or canthopexy may be performed adjunctively to provide additional eyelid support and enhance lid–globe apposition [6,7]. Lateral canthopexy was reported in two cases in this review to improve eyelid positioning [20,28], with temporary tarsorrhaphy [19,23,24] and permanent tarsorrhaphy [7] used to maintain eyelid function and prevent exposure keratopathy in a further four studies. Given the high risk of recurrence due to ongoing scarring and contracture, careful surgical planning, post-operative splinting, and long-term follow-up are essential components of successful management [5]. Interestingly ablative fractional CO2 laser resurfacing was used in two studies included in this scoping review [8]; however, it is not widely discussed in the literature as a surgical intervention to improve scar outcomes and reduce ectropion recurrence.

Non-surgical management of post-burn eyelid ectropion focuses primarily on the prevention of corneal exposure and the mitigation of scar contracture during the acute and subacute phases of healing [5]. Conservative strategies include the application of ocular lubricants such as artificial tears and ophthalmic ointments to maintain corneal hydration and protect against exposure keratopathy [8]. Mechanical interventions, such as temporary eyelid taping [30,31] or the use of external eyelid weights, may assist in facilitating eyelid closure and minimizing lagophthalmos [9]. In some cases, temporary tarsorrhaphy—either adhesive or surgical—can be utilized to partially or fully close the eyelids, thereby protecting the cornea during critical periods of healing [9]. The findings of this scoping review contrast the existing evidence base, with no studies reporting the use of external eyelid weights as a non-surgical technique. Only one study in this review, by Clayton et al. [10], referred to early taping and scar management techniques as a method to address ectropion post burn injuries. However, early and consistent therapy, including gentle massage and scar mobilization, may help to reduce cicatricial contracture and improve tissue elasticity [11]. Early interventions for the prevention of eyelid ectropion post burn focusing on optimizing functional outcomes should be considered, by addressing the impact eyelid dysfunction may have on participation in activities of daily living [11].

### Limitations

A primary limitation of this scoping review is the methodological soundness of the included studies, being case studies and case series. While these study designs offer valuable insights, especially in early stages of clinical investigation, they present several inherent limitations that constrain their contribution to the broader evidence base. One of the primary limitations of case series and case studies is the lack of methodological rigor and the consequent low level of evidence they provide in the hierarchy of clinical research [32]. Unlike randomized controlled trials (RCTs) or well-designed cohort studies, these observational designs lack control groups, randomization, and blinding, which are essential to minimize bias and confounding variables [32]. All studies included in this scoping review consist of case reports or case series with relatively small sample sizes, ranging from one to twenty-six participants. This limitation compromises the validity and generalizability of the findings [33]. Consequently, there is a restricted capacity to draw robust conclusions from the available data or to establish a consensus regarding treatment and prevention strategies for eyelid ectropion within the current literature. Finally, a lack of homogeneity in outcome measures collected limited the potential for meaningful data synthesis across included studies.

## 5. Conclusions

This scoping review highlights the limited and heterogeneous nature of the current evidence regarding the management and prevention of eyelid ectropion following burn injuries. The available literature is predominantly composed of observational studies, including case reports and case series, which reduces the ability to draw strong conclusions about the efficacy of both surgical and non-surgical techniques. Despite consensus on the importance of early intervention to improve functional and cosmetic outcomes, there remains a lack of standardized treatment protocols and clear guidance on optimal management strategies. The review also reveals a significant gap in research addressing non-surgical techniques, with only one study identified in this area, underscoring the need for further exploration. The variability in treatment modalities, and outcome measures further complicates the synthesis of data and limits the generalizability of the findings. Given the functional impairments and psychosocial impacts associated with eyelid ectropion post burn, there is a need for well-designed, larger-scale studies employing standardized methodologies and outcome assessments. Future research should aim to establish evidence-based clinical guidelines to optimize both functional and aesthetic outcomes in this population. Ultimately, enhancing the evidence base will support clinicians in delivering effective, patient-centred care for individuals affected by burn-related eyelid ectropion.

## Figures and Tables

**Table 1:** Scoping review inclusion and exclusion criteria.

Inclusion Criteria	Exclusion Criteria
Age 18 years or older Burn injury/injuries to face including eye(s) Facial burn ≥ superficial partial thickness Involving treatment/intervention for eyelid ectropion Conducted in acute hospital or outpatient department setting	Age younger than 18 years Pre-existing eye conditions impacting function or cosmetic appearance Participants presenting greater than 18 months post burn injury

**Table 2:** Characteristics of studies included in review.

**Study**	**JBI Level**	**Study Design**	**Aim**	Sample * (*n* = Eyes)	**Aim of Intervention for Ectropion**	**Technique**
**Clauss, Bineshfar [7]** 2024 USA	4	Retrospective review	To describe a complex and challenging patient with cicatricial eyelid ectropion and discuss the management principles for eye exposure keratopathy and eyelid retraction therapy.	*n* = 2	Management	Surgical
**Clayton, Haertsch [16]** 2019 Australia	4	Retrospective single-centre case study	To evaluate the efficacy of ablative fractional CO2 laser intervention early in the acute treatment of panfacial burn injury.	*n* = 2	Prevention & Management	Surgical & non-surgical
**Elbanoby, Elbatawy [18]** 2016 Egypt	4	Case series	To present a single pertinent solution to address all problems in the periorbital area.	*n* = 14	Management	Surgical
**Hou, Hou [19]** 2024 China	4	Case series	To retrospectively examine upper and lower eyelid adhesions using an orbicularis oculi muscle flap and verify its stability.	*n* = 46	Management	Surgical
**Jeong, Alessandri-Bonetti [20]** 2024 USA	4	Case series	To describe the complication rates in burn eyelid reconstruction at a single centre for 14 years.	*n* = 23	Management	Surgical
**Jovanovic, Dizdarevic [21]** 2018 Bosnia and Herzegovina	4	Case report	To present a case of bilateral cicatricial eyelid ectropion management following severe burn injuries in a patient who previously sustained severe, deep dermal thermal injuries.	*n* = 2	Management	Surgical
**Keilani, De Faria [17]** 2021 France	4	Case study	To present the case of a woman who presented second- and third-degree burns of the eyelids secondary to physical domestic assault with acid, who had early surgical management with a full-thickness skin graft.	*n* = 2	Prevention & Management	Surgical
**Lee, Levitt [22]** 2018 USA	4	Case report	To report on the efficacy and safety of a novel nonsurgical approach to treating cicatricial ectropion using ablative fractional laser resurfacing and laser-assisted delivery of 5-fluorouracil.	*n* = 1	Management	Surgical
**Lymperopoulos, Jordan [23]** 2016 UK	4	Case report	To describe our early experience of a novel technique for temporary lateral tarsorrhaphy with forehead hitch, which protects the globe and counters the scar- and gravity-related ectropic effects on the lower eyelids.	*n* = 2	Management	Surgical
**Papadopoulou, Nikolaidou [24]** 2023 Greece	4	Case report	To stress the need for preventive measures regarding the use of chemicals and for close observation and timely surgical intervention in chemical burn patients to prevent and limit disfigurement.	*n* = 2	Management	Surgical
**Takaya, Sakai [25]** 2024 Japan	4	Retrospective cohort study	To describe a new technique for correcting contractures and deformities that reliably addresses lacrimal punctum deviation and severe cicatricial lower eyelid ectropion.	*n* = 1	Management	Surgical
**Vana, Isaac [26]** 2014 Brazil	4	Retrospective analysis	To evaluate the outcome of 8 extrinsic ectropion’s secondary to facial burns treated with facial suspension technique.	*n* = 3	Management	Surgical
**Yeşiloğlu, Şirinoğlu [27]** 2014 Turkey	4	Retrospective case series	To present a simple but useful technique involving the V-Y advancement of the eyelid or eyelids in the vertical direction for the prevention of cicatricial ectropion and eyelid contraction.	*n* = 1	Management	Surgical
**Zucal, Waldner [28]** 2022 Switzerland	4	Case report	To present our surgical technique for lateral canthopexy in combination with full-thickness skin grafting in patients with eyelid axis distortion after scar contraction of the periorbital region after severe burn injuries of the face.	*n* = 10	Management	Surgical

* Of eligible participants only.

**Table 3:** Intervention and outcomes for included studies.

**Study**	**Participants**	**Sample (*n* = Eyes)**	**Intervention**	**Outcome Measures**	**Findings**
**Clauss, Bineshfar [7]** 2024	1 patient, 43 years old with 50% TBSA burns including scalp and facial burn	*n* = 2	**Initial management**: multiple procedures including ReCell and Meek micrografting. **42 days post injury**: Partial-thickness autologous skin grafting—integra grafts. **Post-operative day 8**: Bilateral Gunderson Flaps and Synthetic Skin Substitute for Anterior Lamellae Lengthening, and Bilateral permanent Tarsorrhaphies. **42 days post-surgery**: repeat Repair for Recurrent Retraction with Autologous Free FTSG *.	Ectropion recurrence; Lagophthalmos; Eyelid function/competence; Exposure keratopathy.	**Post surgery 1:** Ectropion recurrence—yes Lagophthalmos—present Eyelid function—incompetent Exposure keratopathy—present, bilateral corneal ulcers. **Post surgery 2:** Ectropion recurrence—yes **Post surgery 3:** Ectropion recurrence—nil Lagophthalmos—nil Eyelid function—competent Exposure keratopathy—nil
**Clayton, Haertsch [16]** 2019	1 patient, 39 years old with 68% TBSA burns including facial burn	*n* = 2	**Initial management**: Blunt debridement and Biobrane xenograft applied to burns. **From 48 h post injury:** Nonsurgical orofacial scar contracture management: AROM exercises, stretching, mouth splint, topical lubricant applied frequently for corneal protection. **42 days post injury:** Ablative fractional CO2 laser and non-surgical scar contracture management. 4x sessions over 8 months, 6- to 8-week intervals. **133 days post injury**: Lower eyelid taping, continued daily.	Ectropion resolution; Lagophthalmos resolution; Deficit in eye closure (mm); Eyelid function; Photographs of eye closure at rest and maximal active eye closure.	**Post surgical treatment:** Ectropion—resolved Lagophthalmos—resolved Deficit in eye closure—reduced to 0 mm Eyelid function—competent Photographs—range of motion, eye closure returned to normal at rest and normal active eye closure
**Elbanoby, Elbatawy [18]** 2016	4 patients with chemical burns; 8 patients with thermal burns, 2x bilateral ectropion	*n* = 14	**Initial treatment (Occurred at time of injury in various hospitals):** Unilateral FTSG to release both eyelids *n* = 4; Bilateral FTSG to release both eyelids *n* = 1; STSG * to release lower eyelid *n* = 3 **Later reconstructive treatment’:** Periorbital reconstruction using bifurcated superficial temporal artery island flap (BSTIF). Two patients underwent bilateral periorbital flap reconstruction, 10 patients underwent unilateral reconstruction.	Complications; Eyelid incompetence; Lagophthalmos; Photographs of post-burn scarring	**Post reconstructive surgery:** Complications—Nil Eyelid incompetence/lagophthalmos—reduced to 0 mm in 10 cases, 1–2 mm in 2 cases Repeated procedure—nil Photographs—post-burn scarring appearance reduced.
**Hou, Hou [19]** 2024	26 patients with burns including facial burns	*n* = 46	**Initial treatment:** 6 (9 eyes) had not previously undergone skin grafting or other treatments for eyelid adhesion, while the remaining 20 (37 eyes) had undergone tarsorrhaphy and/or skin grafting after which ectropion recurrence occurred. **Reconstructive procedures**: The tunnel orbicularis oculi muscle flap technique. FTSG was then performed. Average time from burn to reconstructive treatment was 533 days (range 91 to 183 days).	**Average:** Adhesion time; Lagophthalmos/eye exposure; Eyelid closure; Eyelid separation (open eyes); Ectropion recurrence; Adhesion failures; Grafting failure rate (%).	**Last follow up:** Average adhesion time—21.87 months in the 46 eyes Lagophthalmos—resolved Eyelid closure—reduced from 7.72 mm to 0.22 mm Eyelid separation (open eyes)—reduced from 13.89 mm to 8.75 mm Ectropion/contracture recurrence—nil Adhesion failures—nil Grafting failure rate < 2%.
**Jeong, Alessandri-Bonetti [20]** 2024	14 patients with facial burns, average 39.5 ± 19.7% TBSA	*n* = 23	Acute *n* = 10; Acute then reconstructive *n* = 23. **First surgery:** FTSG: *n* =9; Skin substitute and FTSG: *n* = 2 Lateral canthoplasty: *n* = 2; Fractional lasering: *n* = 1; **Second surgery:** FTSG: *n* = 4; Z-plasty: *n* = 1; STSG: *n* = 1 **Third surgery:** Flap and canthoplasty: *n* = 1; Skin substitute and FTSG: *n* = 2	Success rate in group (n/total (%) in correcting eyelid ectropion without recurrence)	**First surgery:** FTSG—33.33% Skin substitute and FTSG—100% Lateral canthoplasty—100% Fractional lasering—50% **Second surgery: **FTSG—50% Z—plasty—100% STSG—100% **Third surgery: **Flap and canthoplasty—100% Skin substitute + FTSG—50%
**Jovanovic, Dizdarevic [21]** 2018	1 patient, 31 years old, 60% TBSA severe deep thermal burns with facial involvement	*n* = 2	**Initial treatment (out-of-country treatment)**—**13 surgeries:** several necrotomies and the Meek Micrografting technique procedures with two repeated keratinocytes cultures harvesting in their tissue bank. **244 days post injury:** bilateral lower eyelid reconstructive surgery—Skin cantus-to-cantus incision, contracture release, orbicularis liberation, and lid elevation; and oversizing free FTSG (Wolfe technique) from the left inguinal region. Residual lower left lid laxity was addressed by pentagonal wedge resection. Decompressive fasciotomy and prolonged treatment for 213 days.	Eyelid closure deficit. Graft take Corneal exposure; Complications	**6 months post-surgery:** Eyelid closure deficit—reduced to mild in lower lid Graft take—100% Corneal exposure—no extensive corneal exposure Complications—nil
**Keilani, De Faria [17]** 2021	1 patient, 43 years old, 8% TBSA deep dermal to full-thickness chemical burns.	*n* = 2	**Treatment 11 days post injury** (to release contracture and prevent ectropion): Upper and lower eyelid excision and FTSG over two procedures. The peri-orbital areas were derma braded. Combined with eye drops. Each surgical procedure included a two-staged procedure (debridement and FTSG).	Eyelid closure; Cosmetic appearance; Lagophthalmos; Exposure-related complications.	**Six months after surgery:** Eyelid closure—full Cosmetic appearance—reported as “satisfying” Lagophthalmos—nil Exposure-related complications—nil
**Lee, Levitt [22]** 2018	1 patient, 29-year-old, extensive facial burns	*n* = 1	**122 days post injury:** Reconstructive—adjunctive intralesional 5-FU (5-fluorouracil) injections and AFLR (ablative fractional laser resurfacing) with laser-assisted delivery of topical 5-FU. Delivered over 4 sessions into the periocular scar tissue.	Ectropion; lagophthalmos; Exposure complications; Skin abnormalities; Cosmetics questionnaire (scar appearance)	Ectropion—resolved Lagophthalmos—resolved Exposure complications—nil Skin abnormalities—improved Questionnaire for cosmetics (scar appearance)—POSAS reduced from 89–26.
**Lymperopoulos, Jordan [23]** 2016	1 patient, 19 years old, 96% TBSA mostly full thickness burns, facial involvement.	*n* = 2	**28 days post injury:** Due to early signs of ectropion with corneal exposure bilaterally, skin grafts were required on both lower lids and right cheek: FTSG to lower eyelid, temporary lateral eyelid tarsorrhaphy with forehead hitch using non-absorbable suture material. Suture kept in for 14 days.	Corneal exposure; Ectropion resolution; Photographs (functional/cosmetic); Eyelid closure	**548 days post-surgery:** Ectropion—resolved Photographs—excellent functional and cosmetic result at 548 days. Eyelid closure—complete Corneal exposure—0 mm
**Papadopoulou, Nikolaidou [24]** 2023	1 patient, 45-year-old with chemical burns; facial involvement, delayed presentation for medical treatment	*n* = 2	**Surgical: Post injury day 60**—FTSG left upper & lower eyelid, FTSG right lower eyelid, partial lateral tarsorrhaphy left. **Day 72**—FTSG right upper eyelid. **Day 146**—Repeat FTSG right lower eyelid. **Day 474**—Tarsorrhaphy release with local flap, Z Plasties, V-Y Plasties to both eyelids and canthal areas. **Between surgeries**, sessions of triamcinolone acetonide intralesional injection were completed to soften specific areas with hypertrophic scarring. **Conservative:** custom-made compressive face mask with silicone sheets	Ectropion recurrence; Eyelid closure; Scar maturation; Eyelid competence; Conservative measure—scar contraction	**Conservative measure:** Unable to prevent scar contraction. **913 days post burn:** Ectropion recurrence—nil Eyelid closure—2/2 eyes satisfactory, adequate & unforced Scar maturation—adequate Eyelid competence—deficit reduced but ongoing need for lubrication
**Takaya, Sakai [25]** 2024	1 patient, 73-year-old with facial burns, recurring ectropion following previous surgical intervention	*n* = 1	**Initial approach:** FTSG, scar revision, and lateral tarsal strip surgery. Scar recurred with lachrymation, inadequate eyelid closure, and lower eyelid ectropion **Reconstructive surgical approach:** Horner Muscle suture and fascia graft—left lower eyelid.	Ectropion; Lagophthalmos; Cosmetic complications; Dry eye symptoms; Retraction	**Post-surgery:** Ectropion—resolved Lagophthalmos—0 mm Cosmetic complications—nil Dry eye symptoms—nil Retraction—5.5 mm
**Vana, Isaac [26]** 2014	2 patients, over 18 years of age, with facial burns	*n* = 3	**Reconstructive/revision post burn (non-acute)** Both patients: Subperiostal suspension. Patient 2: Skin grafting bilateral.	Vertical positioning of eyelid margin; Clinical symptoms; Complications; Need for additional surgeries; Skin graft integration. **Subjective interview:** Clinical symptomatology (yes/no symptom questionnaire); Appearance	**Evaluation 274 days post surgery:** Vertical positioning of the eyelid margin—average 19% Gain * Integration of skin grafts—100% Clinical symptoms (lacrimation, red eyes, and ocular occlusion difficulty)—Good (improvement of 100% of symptoms) Incidence of complications—nil Need for additional surgeries—nil **Subjective interview:** Clinical symptomatology (yes or no questionnaire): Symptoms—moderate (>50%) improvement; Appearance—moderate (>50%) improvement
**Yeşiloğlu, Şirinoğlu [27]** 2014	17 patients with periorbital burns	*n* = 17	**Reconstructive surgical technique:** Vertical lid V-Y advancement technique.	Ectropion; Lagophthalmos; Presence of major complications; Presence of minor complications; Scar appearance	**Post surgical intervention:** Ectropion—nil Lagophthalmos—nil Presence of major complications—nil Presence of minor complications —*n* = 2 minor complications (resolved) Scar appearance—minimal & satisfactory
**Zucal, Waldner [28]** 2022	5 patients, with burn TBSA ranging from 36-88% and facial involvement.	*n* = 10	**Reconstruction 61 to 183 days post injury**: Combined FTSG application and lateral canthopexy. Four of five patients underwent **further interventions** for scar release and FTSG: Canthopexy *n* = 3 from 2 patients, Scar release and FTSG *n* = 7 from 4 patients. **Case 1:** bilateral lower eyelid ectropion and upper eyelid retraction. **152 days post injury:** bilateral ectropion correction with scar release of upper and lower eyelid, supraclavicular FTSG, and bilateral canthopexy. **274 days post injury:** bilateral ectropion recurrence. Revision surgery—scar release and FTSG. **Case 2: 183 days post injury**: bilateral FTSG and lateral canthoplexy to correct bilateral ectropion and eyelid axis distortion. **518 days post injury:** recurrence of ectropion. Scar release and FTSG. **883 days post injury**: re-canthoplasty and additional tarsal strip procedure. **1370 days post injury**: scar release and FTSG. **Case 3: 91 days post injury**: functional correction of the bilateral ectropion with scar release followed by FTSG and lateral canthopexy. **274 days post injury**: correction of medial ectropion with medial canthopexy and z-plasty **Four years later**, another surgical correction and FTSG for medial lower ectropion. **Case 4: 61 days post injury**: Scar release of upper and lower eyelid, FTSG from cervical area, and lateral canthopexy. **Case 5: 183 days post injury:** bilateral ectropion of the lower eyelids. Scar release, FTSG (from the right groin), and lateral canthopexy. **274 days post injury**: FTSG repeated for recurrent scar contraction. **457 days post injury:** re-canthopexy (bilaterally) with scar release and FTSG.	Symmetry; Eyelid closure; Complications; Lagophthalmos and eyelid closure; Recurrence.	**Surgical follow up (median 487 (61 to 122 days):** Symmetry—improved in all 5 patients Eyelid closure—forced closure restored in 5/5 patients. Complete relaxed eyelid closure bilaterally in 2 patients, complete relaxed closure unilaterally in another 2 patients. Forced closure bilaterally in 1 patient. Complications—nil Exposure symptoms—resolved or reduced Lagophthalmos—reduced to 0–3 mm; in 1 case, there was a reduced but persistent bilateral lagophthalmos (1.5 mm on the right and 3.0 mm on the left), with complete forced closure. Recurrence—surgical revision required *n* = 2 (recurrence of unilateral lower eyelid retraction).

Key: FTSG—Full-thickness skin graft; STSG—Split-thickness skin graft; POSAS—The Patient and Observer Scar Assessment Scale: AROM—Active range of motion.

## Appendix A

### Appendix A.1. PRISMA-ScR Checklist

**Table A1:** Preferred Reporting Items for Systematic reviews and Meta-Analyses extension for Scoping Reviews (PRISMA-ScR) checklist.

SECTION	ITEM	PRISMA-ScR CHECKLIST ITEM	REPORTED ON PAGE #
TITLE			
Title	1	Identify the report as a scoping review.	1
ABSTRACT			
Structured summary	2	Provide a structured summary that includes (as applicable): background, objectives, eligibility criteria, sources of evidence, charting methods, results, and conclusions that relate to the review questions and objectives.	2
INTRODUCTION			
Rationale	3	Describe the rationale for the review in the context of what is already known. Explain why the review questions/objectives lend themselves to a scoping review approach.	4
Objectives	4	Provide an explicit statement of the questions and objectives being addressed with reference to their key elements (e.g., population or participants, concepts, and context) or other relevant key elements used to conceptualize the review questions and/or objectives.	5
METHODS			
Protocol and registration	5	Indicate whether a review protocol exists; state if and where it can be accessed (e.g., a Web address); and if available, provide registration information, including the registration number.	5
Eligibility criteria	6	Specify characteristics of the sources of evidence used as eligibility criteria (e.g., years considered, language, and publication status), and provide a rationale.	5, 6
Information sources*	7	Describe all information sources in the search (e.g., databases with dates of coverage and contact with authors to identify additional sources), as well as the date the most recent search was executed.	6
Search	8	Present the full electronic search strategy for at least 1 database, including any limits used, such that it could be repeated.	6
Selection of sources of evidence†	9	State the process for selecting sources of evidence (i.e., screening and eligibility) included in the scoping review.	7
Data charting process‡	10	Describe the methods of charting data from the included sources of evidence (e.g., calibrated forms or forms that have been tested by the team before their use, and whether data charting was done independently or in duplicate) and any processes for obtaining and confirming data from investigators.	7
Data items	11	List and define all variables for which data were sought and any assumptions and simplifications made.	47
Critical appraisal of individual sources of evidence§	12	If done, provide a rationale for conducting a critical appraisal of included sources of evidence; describe the methods used and how this information was used in any data synthesis (if appropriate).	8
Synthesis of results	13	Describe the methods of handling and summarizing the data that were charted.	8
RESULTS			
Selection of sources of evidence	14	Give numbers of sources of evidence screened, assessed for eligibility, and included in the review, with reasons for exclusions at each stage, ideally using a flow diagram.	8
Characteristics of sources of evidence	15	For each source of evidence, present characteristics for which data were charted and provide the citations.	10
Critical appraisal within sources of evidence	16	If done, present data on critical appraisal of included sources of evidence (see item 12).	10
Results of individual sources of evidence	17	For each included source of evidence, present the relevant data that were charted that relate to the review questions and objectives.	11–15, 17–28
Synthesis of results	18	Summarize and/or present the charting results as they relate to the review questions and objectives.	16
DISCUSSION			
Summary of evidence	19	Summarize the main results (including an overview of concepts, themes, and types of evidence available), link to the review questions and objectives, and consider the relevance to key groups.	29
Limitations	20	Discuss the limitations of the scoping review process.	32
Conclusions	21	Provide a general interpretation of the results with respect to the review questions and objectives, as well as potential implications and/or next steps.	32, 33
FUNDING			
Funding	22	Describe sources of funding for the included sources of evidence, as well as sources of funding for the scoping review. Describe the role of the funders of the scoping review.	34

### Appendix A.2. Search Strategy

**Table A2:** Search strategy of each database.

Database Search Conducted on 12 August 2024	Search Search Strategy Includes Concepts and Limits: (Eyelid) AND (Ectropion) AND (Burn)	Search Result (*n*) With Date Limit: Scope of Search was Refined to 2014–2024 for Each of the Databases Searched
PubMed search, includes MeSH	(“Ectropion”[Mesh] OR “ectropion”[tiab] OR “ectropions”[tiab]) AND (“Eyelids”[Mesh] OR “eyelids”[tiab] OR “eyelid”[tiab]) AND (“Burns”[Mesh] OR “Eye Burns”[Mesh] OR “Burn Units”[Mesh] OR “burn”[tiab] OR “burns”[tiab] OR “postburn”[tiab] OR “postburns”[tiab] OR “post-burn”[tiab] OR “post-burns”[tiab])	30
Embase, includes Emtree	(‘ectropion’/exp “ectropion”:ti,ab OR “ectropions”:ti,ab) AND (‘eyelid’/exp OR “eyelids”:ti,ab OR “eyelid”:ti,ab) AND (‘burn’/exp OR ‘eye burn’/exp OR ‘burn unit’/exp OR “burn”:ti,ab OR “burns”:ti,ab OR “postburn”:ti,ab OR “postburns”:ti,ab OR “post-burn”:ti,ab OR “post-burns”:ti,ab)	40
CINAHL Complete, includes CINAHL Subject Headings	(TI(“ectropion” OR “ectropions”) OR AB(“ectropion” OR “ectropions”)) AND (MH “Eyelids+” OR TI(“eyelids” OR “eyelid”) OR AB(“eyelids” OR “eyelid”)) AND (MH “Burns+” OR MH “Burn Units” OR MH “Burn Patients” OR TI(“burn” OR “burns” OR “postburn” OR “postburns” OR “post-burn” OR “post-burns”) OR AB(“burn” OR “burns” OR “postburn” OR “postburns” OR “post-burn” OR “post-burns”))	6
Cochrane Library, includes MeSH	ID Search Hits #1 MeSH descriptor: [Ectropion] explode all trees 14 #2 (“ectropion” OR “ectropions”):ti,ab,kw 97 #3 #1 OR #2 97 #4 MeSH descriptor: [Eyelids] explode all trees 1409 #5 (“eyelids” OR “eyelid”):ti,ab,kw 2638 #6 #4 OR #5 3535 #7 MeSH descriptor: [Burns] explode all trees 2439 #8 MeSH descriptor: [Eye Burns] explode all trees 33 #9 MeSH descriptor: [Burn Units] explode all trees 55 #10 (“burn” OR “burns” OR “postburn” OR “postburns” OR “post-burn” OR “post-burns”):ti,ab,kw 6605 #11 #8 OR #9 OR #10 6605 #12 #3 AND #6 AND #11 1	1
SCOPUS search, includes phrase searching and field searching:	TITLE-ABS-KEY((“Ectropion” OR “ectropion” OR “ectropions”) AND (“Eyelids” OR “eyelids” OR “eyelid”) AND (“Burns” OR “Eye Burns” OR “Burn Units” OR “burn” OR “burns” OR “postburn” OR “postburns” OR “post-burn” OR “post-burns”))	49
